# The Effect of SO_2_ on C_3_H_8_ Oxidation over Ru@CoMn_2_O_4_ Spinel

**DOI:** 10.3390/molecules30214253

**Published:** 2025-10-31

**Authors:** Yan Cui, Zequan Zeng, Yaqin Hou, Shuang Ma, Jieyang Yang, Jianfeng Zheng, Wenzhong Shen, Zhanggen Huang

**Affiliations:** 1 State Key Laboratory of Coal Conversion, Institute of Coal Chemistry, Chinese Academy of Sciences, Taiyuan 030001, China; cuiyan@sxicc.ac.cn (Y.C.); zengzequan@sxicc.ac.cn (Z.Z.); houyaqin@sxicc.ac.cn (Y.H.); mashuang20@mails.ucas.ac.cn (S.M.); yangjy@sxicc.ac.cn (J.Y.); zhengjf@sxicc.ac.cn (J.Z.); 2 University of Chinese Academy of Sciences, Beijing 100049, China

**Keywords:** Ru@CoMn_2_O_4_, C_3_H_8_ oxidation, SO_2_ poisoning mechanism, MnSO_4_

## Abstract

Propane is a typical volatile organic compound (VOC) in coal chemical processing and petroleum refining. However, coexisting SO_2_ significantly impairs its catalytic oxidative removal, potentially causing catalyst poisoning and deactivation. This study systematically elucidated the inhibitory effects of SO_2_ on the catalytic oxidation of propane over the Ru@CoMn_2_O_4_ catalyst system. Under continuous exposure to 30 ppm SO_2_, propane conversion plummeted by 30% within two hours. Mechanistic studies revealed that SO_2_ selectively bound to high-valent Mn sites rather than preferentially interacting with Co sites, leading to the formation of MnSO_4_ particles. These particles were directly corroborated by X-ray diffraction (XRD) and transmission electron microscopy (TEM) analyses. After four hours of exposure to SO_2_, roughly 11.8 mole percent of manganese in the catalyst was converted into MnSO_4_. These deposits physically blocked active sites, reduced specific surface area, and disrupted redox cycling. As a result, their combined effects diminished performance progressively, ultimately leading to complete deactivation. Furthermore, in situ diffuse reflectance infrared Fourier transform spectroscopy (DRIFTS) confirmed that SO_2_ suppressed C=C bond oxidation in propane intermediates, thereby directly limiting conversion efficiency. Combining qualitative and quantitative methods, we characterized SO_2_-induced poisoning during propane oxidation. This work provides guidelines and strategies for designing anti-sulfur catalysts at the elemental scale for the catalytic combustion of low-carbon alkanes.

## 1. Introduction

Industrial processes including petroleum refining, coking, and leather manufacturing generate off-gases containing trace quantities of propane [1,2]. As a prototypical VOC, propane presents non-negligible environmental and health hazards. Consequently, research on its catalytic oxidation represented an interdisciplinary endeavor aligned with global ‘dual carbon’ initiatives, simultaneously targeting waste valorization and pollution mitigation. This field held significance not only for addressing industrial exhaust challenges but also for propelling innovation in low-carbon chemical engineering through novel catalyst development, thereby establishing a foundational framework for advanced VOC treatment. However, the inherent stability of propane’s C–H bonds imposed stringent requirements for high reaction temperatures [3,4]. At low temperatures, the high activation energy barrier posed a significant scientific challenge [5,6,7]. Therefore, the development of efficient and environmentally friendly low-temperature catalysts has remained at the core of catalytic oxidation technology.

The oxidation of propane employs common catalysts such as noble metals and transition metal oxides [8,9,10,11,12]. Prior research focused predominantly on optimizing these systems to improve reaction performance [2,13,14]. However, in industrial contexts like coal chemical engineering and petroleum refining, low-concentration propane streams frequently contain coexisting sulfurous pollutants (notably SO_2_). Consequently, understanding the influence of SO_2_ on catalytic propane oxidation assumes significant importance. Investigations into SO_2_ effects have primarily utilized noble metal catalysts (e.g., Pd, Pt) [15,16,17], revealing contradictory findings. Most studies indicate adverse poisoning effects. For instance, Gremminger [18] demonstrated that sulfate formation accelerates deactivation in Pd-based systems. Arevalo [19] proposed a mitigating mechanism whereby SO_2_ is initially oxidized by PdO to SO_3_, which adsorbs onto both the metal and support surfaces, alleviating the complete poisoning of PdO. Conversely, a minority perspective suggests beneficial roles for SO_2_. Chen identified SO_2_-induced surface acidification facilitating propane C-C bond activation and thus boosting reactivity [20]. Ding [3] proposed that SO_2_ pretreatment could increase the oxygen vacancies on the surface of the catalyst and maintain the dynamic equilibrium of SO_2_ adsorption and desorption on the surface, thereby preventing the formation of bulk sulfates.

In recent years, noble metals deposited on spinel supports have garnered significant attention as promising catalysts for alkane oxidation reactions. Although the aforementioned studies have provided valuable insights into the impact of SO_2_ on propane catalytic oxidation, there still lacks a systematic analysis of key scientific issues such as the effects of SO_2_ and its surface reaction pathways on this newly popular class of noble metal-spinel catalysts. Accordingly, extending our prior work [21], this study aims to comprehensively investigate the impact of SO_2_ on Ru@CoMn_2_O_4_-catalyzed propane oxidation across multiple facets: surface morphology evolution, elemental valence state transitions, and reaction intermediate identification. By elucidating the poisoning mechanism of SO_2_ at active sites (including noble metal particles or metal oxide phases), we seek to resolve existing knowledge gaps regarding sulfur interference in heterogeneous catalysis. These findings will establish a theoretical framework for designing robust catalysts capable of operating effectively under complex waste gas conditions.

## 2. Results and Discussion

### 2.1. Catalytic Performance

**Figure 1:**
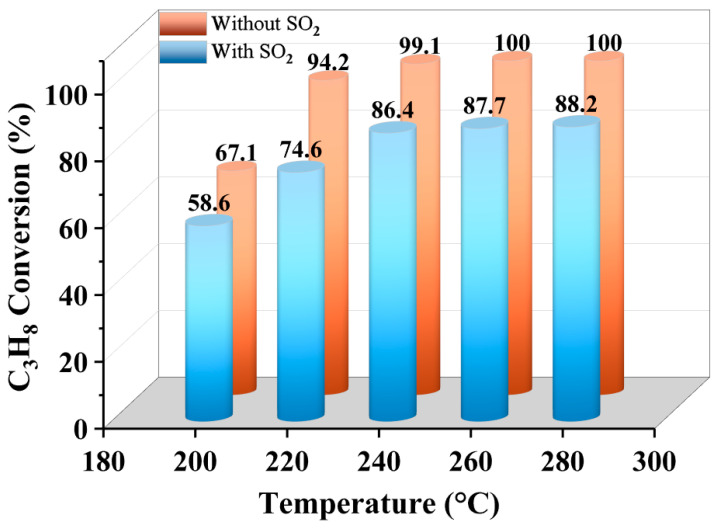
The effect of reaction temperature on the removal of propane over Ru@CoMn_2_O_4_ catalyst (reaction conditions: [C_3_H_8_] = 2000 ppm, [SO_2_] = 30 ppm, 10% O_2_, N_2_ as the balance gas, GHSV = 60,000 mL g^−1^ h^−1^).

The impact of introducing 30 ppm SO_2_ on the catalytic oxidation of propane was evaluated across a range of temperatures. As illustrated in Figure 1, without SO_2_ present, propane conversion increased consistently as temperature increased. When reaching 220 °C, conversion had surpassed 90%; thereafter, the rate of increase diminished progressively as the reaction approached completion. Following SO_2_ introduction, propane conversion decreased at all measured temperatures, demonstrating its inhibitory effect on the reaction. Notably, at 220 °C, SO_2_ caused the most substantial suppression in conversion (≈20% reduction). This pronounced effect likely resulted from significant alterations to the catalyst’s physicochemical properties at this temperature which maximally impeded propane oxidation. Consequently, subsequent investigations focused on SO_2_-induced changes in catalyst textural characteristics and their influence on propane oxidation at 220 °C.

**Figure 2:**
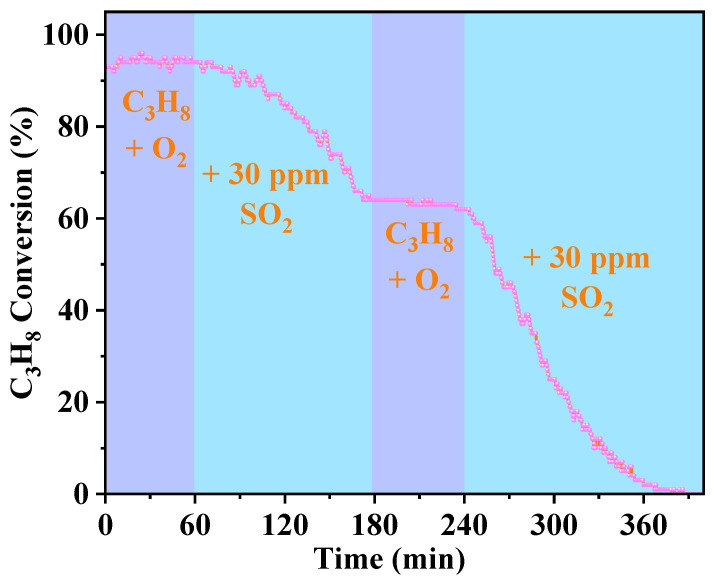
Effect of SO_2_ on propane removal over Ru@CoMn_2_O_4_ catalyst at 220 °C. (Reaction conditions: [C_3_H_8_] = 2000 ppm, [SO_2_] = 30 ppm, 10% O_2_, N_2_ as balance gas, GHSV = 60,000 mL g^−1^ h^−1^).

To further investigate the deactivation of the catalyst in the presence of SO_2_, transient experiments were conducted at 220 °C through periodic introduction and interruption of an SO_2_ stream during propane oxidation (Figure 2). Before exposing the system to SO_2_, propane conversion remained consistently above 90%. When 30 ppm SO_2_ was introduced, conversion initiated a gradual decline, reaching a 30% decrease within two hours. Crucially, after removing SO_2_ from the feed gas, no recovery of catalytic activity occurred. Subsequent reintroduction of SO_2_ triggered a sharp and accelerated activity drop. By six hours into the reaction, activity had nearly completely disappeared. These findings confirm that SO_2_ imposes an irreversible poisoning effect on the catalyst. Moreover, extended contact time between SO_2_ and the catalyst correlated with progressive loss of propane oxidation activity, ultimately leading to complete deactivation.

### 2.2. Physical Characteristics

**Figure 3:**
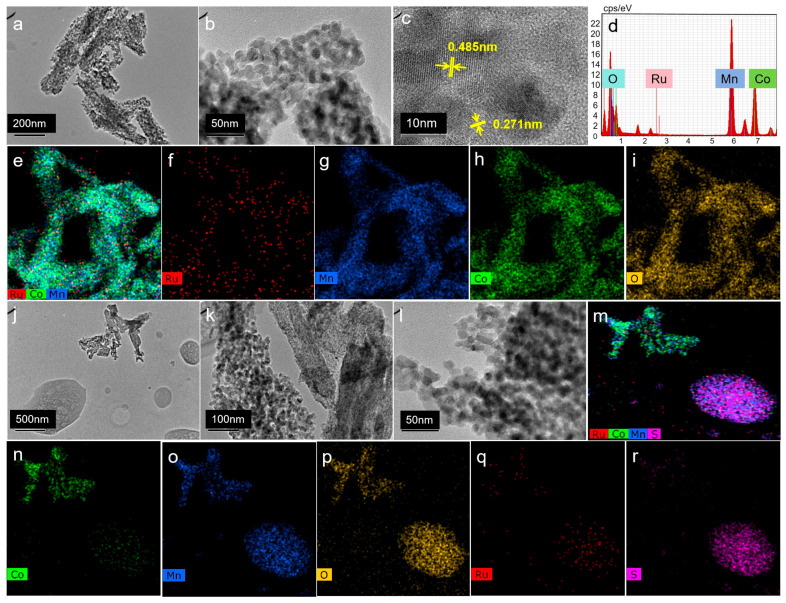
TEM and EDS diagrams of fresh catalyst (**a**–**i**) and poisoned catalyst (**j**–**r**).

To elucidate the origin of SO_2_ poisoning effects on catalytic activity, we initially characterized and compared the structural features of fresh and poisoned catalyst samples. TEM characterization results are presented in Figure 3. As shown in Figure 3a–c, the fresh catalyst consists predominantly of quasi-spherical nanoparticles (approximate 10 nm diameter) exhibiting well-resolved lattice fringes. Lattice d-spacing measurements yielded values of 0.485 nm and 0.271 nm, corresponding to the (111) and (113) crystallographic planes of cobalt manganese spinel, respectively. Energy-dispersive X-ray spectroscopy (EDS) mapping shown in Figure 3e–i confirmed uniform distribution of Ru, Co, Mn, and O throughout the fresh catalyst structure. In contrast, TEM images of the SO_2_-poisoned catalyst [Figure 3j–l] revealed significant morphological changes: coexisting with spherical particles analogous to those in the fresh sample, large ellipsoidal species exceeding 100 nm in diameter were observed. Corresponding EDS analyses demonstrated distinct elemental composition between these two phases. While the spherical aggregates retained uniform distribution of Ru, Co, Mn, and O, the bulk ellipsoidal regions were predominantly composed of Mn, O, and S. Based on these observations, we propose that manganese sulfate formed on the surface of the poisoned catalyst during exposure to SO_2_.

**Figure 4:**
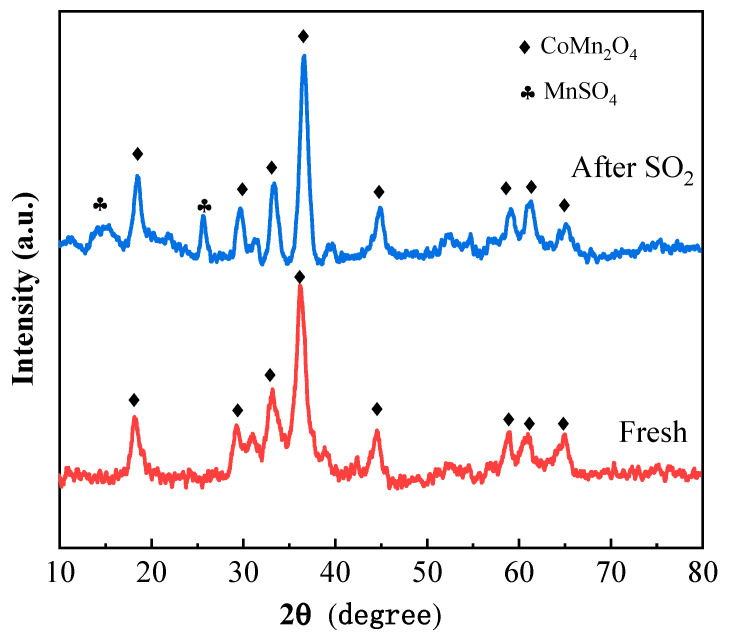
XRD patterns of fresh and poisoned catalysts.

Pore structural parameters were comparatively analyzed for both fresh and SO_2_-poisoned catalyst samples. The data were listed in Table 1. The fresh catalyst exhibited a specific surface area of 82.97 m^2^/g and a pore volume of 0.36 cm^3^/g, characterized by predominantly mesoporous features. Following exposure to SO_2_, these values decreased by 24% and 19%, respectively. This reduction was attributed to the formation of deposits on the catalyst surface, which block pores and consequently reduce accessible surface area and void volume. Meanwhile, the average pore diameter increased slightly, likely due to partial pore widening caused by this blockage effect. It has been reported that crystallization and deposition of sulfates on the catalyst surface or within its pores directly block micropores and some mesopores, leading to a reduction in effective pore size. However, when these pores were blocked, reactants might be forced to diffuse through larger pores, which indirectly resulted in an increase in the apparent pore diameter [22]. To elucidate structural changes at the crystalline level, XRD was utilized. As depicted in Figure 4, the XRD pattern of the fresh catalyst aligns with the reference pattern for cobalt manganese spinel (PDF #77-0741). Notably, no distinct Ru diffraction peaks were observed, presumably owing to its low loading and homogeneous dispersion that prevented long-range order. In contrast, the pattern for the poisoned catalyst revealed additional peaks at 2θ angles of 25.6° and 13.7°, corresponding to the (120) and (110) planes of MnSO_4_ (PDF #35-0751), respectively. What’s more, the characteristic peaks of the CoMn_2_O_4_ spinel exhibited peak shifts towards higher angles. For instance: The (211) plane shifted from 36.3° to 36.8°; The (103) plane shifted from 33.0° to 33.5°; The (101) plane shifted from 18.2° to 18.6°. This phenomenon was likely attributed to the formation of species such as MnSO_4_ on the catalyst surface, which exerted compressional stress on the CoMn_2_O_4_ lattice. Consequently, the interplanar spacing of the CoMn_2_O_4_ spinel reduced, resulting in an increase in the corresponding 2θ values. In summary, the observed morphological evolution and crystallographic transformation provided compelling evidence supporting the preliminary hypothesis that manganese sulfate species formed on the catalyst surface during SO_2_ poisoning.

### 2.3. Surface Chemical Properties

**Figure 5:**
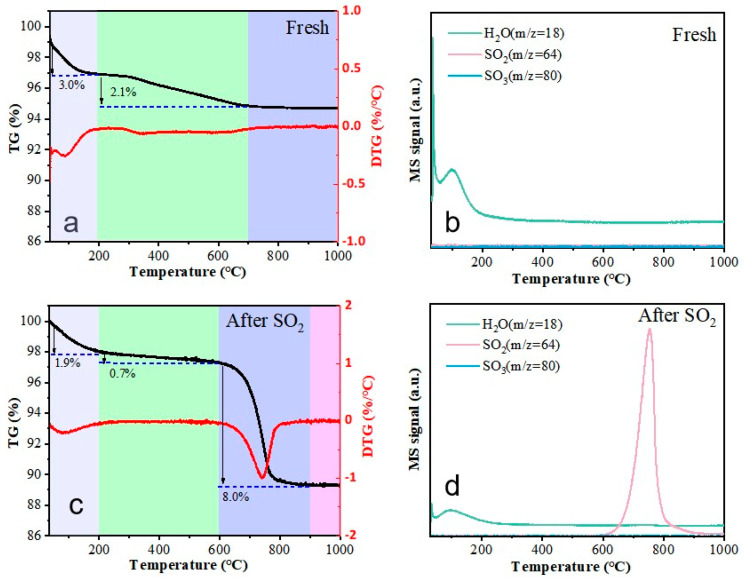
TG curves and MS signal of fresh (**a**,**b**) and poisoned catalysts (**c**,**d**).

Thermogravimetric analysis coupled with mass spectrometry (TG-MS) was utilized to investigate thermal stability and volatilization products for both fresh and SO_2_-poisoned catalyst samples. As shown in Figure 5a,c, the total mass loss over the temperature ramp from 50 °C to 1000 °C amounted to 5.1% for the fresh catalyst and significantly higher at 10.6% for the poisoned counterpart. For the fresh sample, decomposition occurred predominantly below 700 °C: a 3.0% loss between 50 and 200 °C corresponded to adsorbed water desorption; an additional 2.1% reduction between 200 and 700 °C presumably originated from surface carbonaceous deposits. No further mass change was observed above 700 °C. In contrast, the poisoned catalyst exhibited distinct behavior, featuring a pronounced weight loss event between 600 and 900 °C (peaking at 741 °C on the derivative thermogravimetric (DTG) curve) accounting for approximate 8.0% of its initial mass (Figure 5c). Correlation with corresponding MS spectra (Figure 5b,d) identified intense SO_2_ release coincident with the high-temperature degradation step. Previous studies [23,24,25,26] have established that MnSO_4_ decomposes within 600–800 °C (via 2MnSO_4_(s) → 2MnO(s) + 2SO_2_(g) + O_2_(g)) and CoSO_4_ undergoes desulfurization only above 950 °C (via 3CoSO_4_(s) → Co_3_O_4_(s) + 3SO_2_(g) + O_2_(g)). The above-mentioned SO_2_ release peak unambiguously indicates MnSO_4_ decomposition rather than CoSO_4_ breakdown. This finding provided direct experimental validation for our hypothesis regarding manganese sulfate formation on the poisoned catalyst surface. Furthermore, based on the mass loss above 600 °C, we calculated that approximately 3.02 mg of MnSO_4_ had been generated from 20 mg of the catalyst. This indicates that after four hours of exposure to SO_2_ (30 ppm), roughly 11.8 mole percent of manganese in the catalyst had been converted into MnSO_4_.

**Figure 6:**
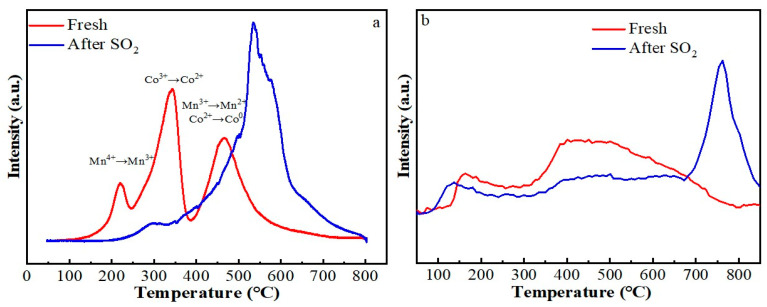
H_2_-TPR (**a**) and O_2_-TPD (**b**) of fresh and poisoned catalysts.

Hydrogen temperature-programmed reduction (H_2_-TPR) analyses were conducted to evaluate alterations in redox properties induced by SO_2_ poisoning of the catalyst (Figure 6a). The fresh catalyst exhibited three distinct reduction stages: reduction of Mn^4+^ to Mn^3+^ commencing at approximately 220 °C [27], conversion of Co^3+^ to Co^2+^ centered at 342 °C; and subsequent parallel reductions of Mn^3+^ to Mn^2+^ and Co^2+^ to metallic Co (Co^0^) peaking at 466 °C [28,29]. Upon exposure to SO_2_, the reduction temperature of Mn^4+^ to Mn^3+^ increased from 220 °C to 300 °C, accompanied by a pronounced decline in peak intensity. Notably, a new H_2_ consumption band appeared in the poisoned sample within the 500–600 °C range. In conjunction with TG-MS data, the H_2_ consumption peak was attributed to the reduction of MnSO_4_ formed upon SO_2_ poisoning of the catalyst. This assignment aligns with findings from relevant literature studies [30,31]. Due to the dominance of sulfate-derived peaks, the reduction peaks of Mn^3+^→Mn^2+^, Co^3+^→Co^2+^, and the formed sulfates merged into a broad composite peak within the temperature range of 350–700 °C. Although precise quantitative analysis of H_2_ consumption remained challenging, we propose that following SO_2_ poisoning, both the reduction peaks of Mn^3+^→Mn^2+^ and Co^3+^→Co^2+^ exhibited a pronounced shift toward higher temperatures, demonstrative of significant suppression in the redox property.

Oxygen species represent a key determinant of catalytic redox performance. To investigate changes in these species induced by poisoning, O_2_-temperature programmed desorption (O_2_-TPD) analyses were performed on both fresh and SO_2_-poisoned samples (Figure 6b). According to established interpretations [27,32], low-temperature desorption peaks below 300 °C correspond to weakly held surface oxygen species (including molecular O_2_, superoxide O_2_^−^, and peroxide O^−^), whereas features within the 300–600 °C range originate from lattice oxygen (O^2−^). Prior investigations [21] have established that while surface-adsorbed oxygen exhibits low reactivity and contributes minimally to propane oxidation, lattice oxygen acts as the dominant active component. As evidenced by Figure 6b, the fresh catalyst exhibited a significantly greater abundance of lattice oxygen compared to adsorbed species. In contrast, exposure to SO_2_ caused a severe reduction in lattice oxygen, closely paralleling its reduced activity. A notable peak emerged above 700 °C, observed only for the poisoned sample. As documented in the literature, this high-temperature feature corresponds to the thermal decomposition of metal sulfates [33].

**Figure 7:**
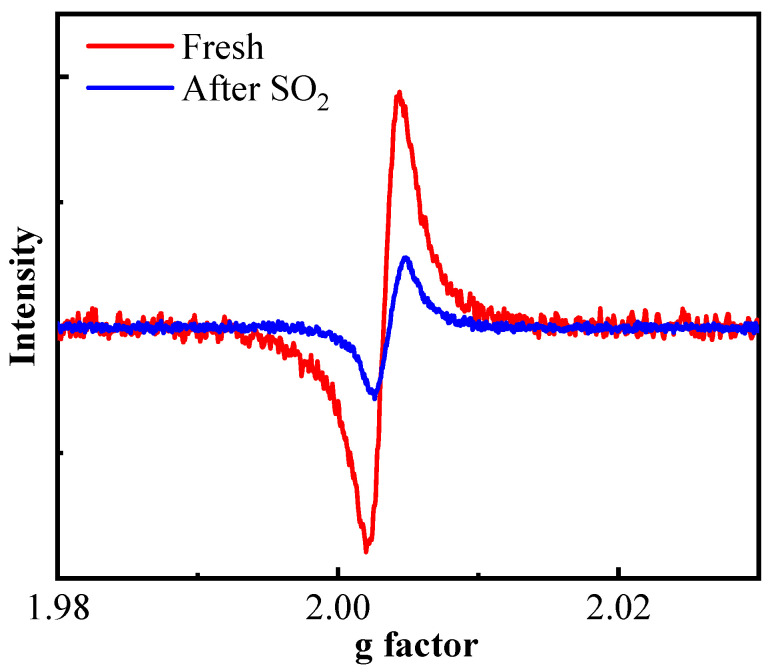
EPR spectra of fresh and poisoned catalysts.

Oxygen vacancies are also significant indicators for characterizing the redox performance of catalysts. We further conducted Electron paramagnetic resonance (EPR) spectroscopy on the fresh and poisoned catalysts to determine the content of oxygen vacancies (Figure 7). All spectra displayed a prominent isotropic signal at g = 2.003, whose intensity correlated linearly with the density of oxygen vacancies [33]. Notably, the poisoned catalyst exhibited a substantial reduction in signal intensity compared to its fresh counterpart, demonstrating that sulfur exposure suppressed oxygen vacancy formation. Given that oxygen vacancies facilitate lattice oxygen mobility and reactivity, the decrease consequently impaired the activity of lattice oxygen species. These findings are fully consistent with those obtained from O_2_-TPD analysis.

**Figure 8:**
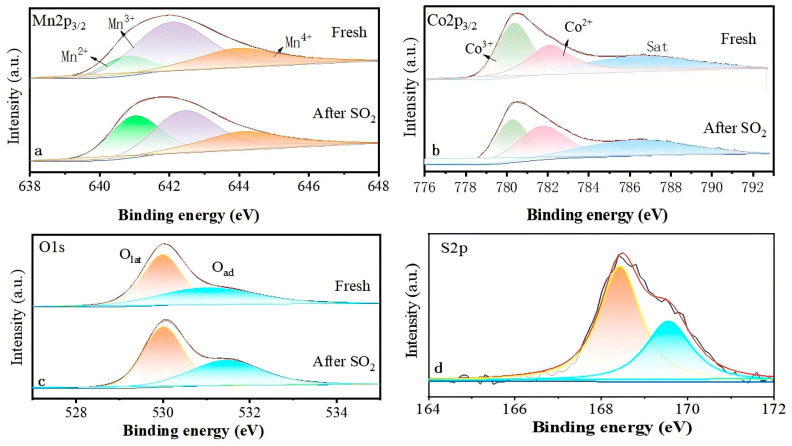
XPS spectra of (**a**) Mn 2p, (**b**) Co 2p, (**c**) O 1s, (**d**) S 2p of fresh and poisoned catalysts.

The surface chemical states of both fresh and SO_2_-poisoned catalysts were characterized by X-ray photoelectron spectroscopy (XPS). High-resolution scans of the Mn 2p_3/2_ region (Figure 8a) identified signals corresponding to Mn^2+^ (640.8–641.0 eV), Mn^3+^ (642.0–642.4 eV), and Mn^4+^ (644.0–644.1 eV) based on literature assignments [8,11,34]. Peak area integration provided the relative abundances summarized in Table 2. In the fresh catalyst, Mn^3+^ constituted the majority species, accompanied by lower amounts of Mn^4+^ and minimal Mn^2+^. Following SO_2_ exposure, significant decreases were observed in both Mn^3+^ and Mn^4+^, while the Mn^2+^ content increased markedly—approximately 2.4-fold compared to its initial level. The transformation corroborated complementary TEM, XRD, and TG-MS findings, collectively supporting a mechanism where gaseous SO_2_ reacted with surface Mn sites to form MnSO_4_. This reaction depleted high-oxidation-state manganese species, directly impairing the material’s redox capacity and leading to irreversible catalyst deactivation. Figure 8b shows the high-resolution spectrum of Co 2p_3/2_. The peaks at 780.3–780.4 eV, 782.1–782.2 eV, and 786.4–786.5 eV were attributed to Co^3+^, Co^2+^ and the strong satellite peak associated with Co^2+^, respectively [35]. Analogous to the Mn trend, the surface valence distribution of Co shifted towards lower oxidation states post-poisoning. Although no distinct CoSO_4_ phase was detected via XRD, TEM, or TG-MS analyses after SO_2_ exposure, XPS results revealed partial reduction of Co^3+^ to Co^2+^. This decline in redox capability represented one contributing factor to the decrease in propane oxidation activity.

The O1s spectrum typically resolves into two components: lattice oxygen (O_lat_) at 529.9–530.0 eV and chemically adsorbed oxygen (O_ad_) at 531.0–531.5 eV [36]. Deconvolution analysis of the spectra presented in Figure 8c, with quantitative results listed in Table 1, revealed a decrease in the O_lat_/O_ad_ integrated intensity ratio from 1.44 for the fresh catalyst to 1.33 after SO_2_-poisoning. This ratio serves as a key indicator of lattice oxygen availability, which is crucial for promoting propane oxidation kinetics. Upon SO_2_ poisoning, the relative content of lattice oxygen decreased, directly impacting catalytic activity. Additionally, in the poisoned catalyst, the peaks of O_lat_ and O_ad_ shifted towards higher binding energies, indicating that the formed sulfate influenced the coordination environment of the O element in the catalyst. A higher binding energy made it more challenging for oxygen species to participate in the reaction, thus reducing the catalytic activity.

In the S2p spectrum of the poisoned catalyst (Figure 8d), two distinct peaks were observed. The lower binding energy peak at 168.4 eV was assigned to tetravalent sulfur (S^4+^), indicative of molecularly adsorbed SO_2_, whereas the higher energy peak at 169.5 eV corresponded to hexavalent sulfur (S^6+^) in either sulfate or sulfite forms [37,38]. These spectral features confirmed significant SO_2_ adsorption followed by its irreversible transformation into stable sulfur-containing compounds. Such chemical transformation provided direct evidence supporting the proposed deactivation mechanism attributed to sulfur poisoning.

### 2.4. Poisoning Mechanism

**Figure 9:**
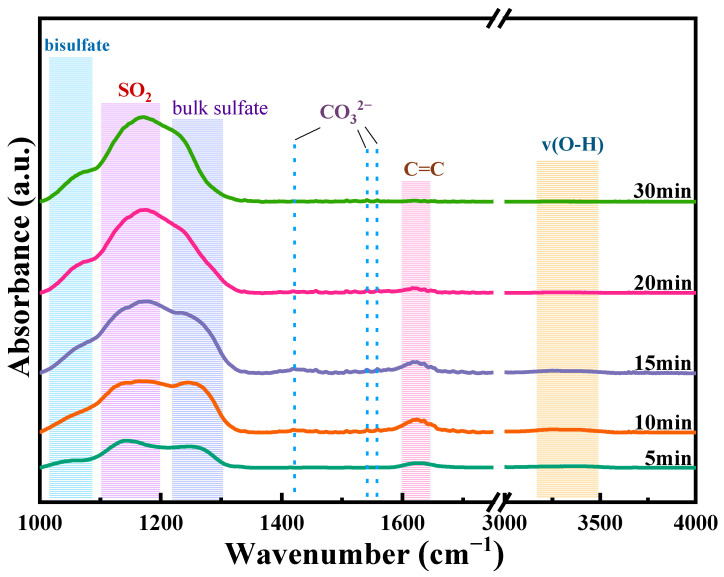
Effect of SO_2_ on the catalytic oxidation of propane at different reaction times.

To investigate the mechanism of SO_2_-induced deactivation during propane total oxidation over Ru@CoMn_2_O_4_, in situ DRIFT spectroscopy was utilized to monitor the evolution of reaction intermediates. As shown in Figure 9, the acquired spectra revealed changes associated with progressive surface sulfation. Based on literature assignments, key bands were identified: (i) A broad envelope between 3200 and 3500 cm^−1^ corresponds to hydrogen-bonded hydroxyl groups in molecularly adsorbed water layers [9,11]; (ii) Three resolved peaks at 1422, 1542, and 1560 cm^−1^ constitute the characteristic fingerprint of amphoteric carbonate species, attributed to bicarbonate/resonance structures formed during redox cycling of propane fragments [10,11,39]; (iii) An isolated absorbance at 1622 cm^−1^ is assigned to localized C=C double bond stretching vibrations within partially dehydrogenated hydrocarbon intermediates adsorbed on coordinatively unsaturated metal centers [39]; (iv) Three distinct sulfur-containing species emerge at higher wavenumbers—mondentate M-OSO_3_^−^ linkages (bridging mode) near 1056 cm^−1^, weakly bound molecular SO_2_ at 1146 cm^−1^, and polymeric bulk sulfate networks around 1248 cm^−1^ [30,33,40]. Upon introducing SO_2_ into the reactor feed stream, significant dynamic responses were observed: (a) The molecular SO_2_ band intensified and underwent a systematic blueshift from its initial position at 1146 cm^−1^ toward 1170 cm^−1^; (b) Both monodentate and bulk sulfate features amplified monotonically; (c) All carbonate vibrational modes were rapidly quenched within minutes of SO_2_ exposure. As the contact time between SO_2_ and the catalyst extended, the cumulative adsorption quantity of SO_2_ on the catalyst surface progressively increased, thereby enhancing the intensity of its molecular characteristic peaks. The observed peak position shift was likely attributed to the chemical reaction between surface-adsorbed SO_2_ and the catalyst, resulting in sulfate formation. This newly formed species altered the infrared spectral position of SO_2_ peaks. Simultaneously, the olefinic C=C stretching band exhibited non-linear behavior: initial exponential growth reaching maximum intensity at moderate sulfate coverage followed by abrupt decay. This trend suggests that the sulfate species covered the active sites, inhibiting the cleavage of the C=C bond in the propane intermediate and preventing further deep oxidation, resulting in the accumulation of C=C. When the catalyst was completely deactivated, the C=C peak vanished. These converging lines of evidence demonstrated that extensive bulk sulfate formation occurred universally under SO_2_ exposure. This process was definitively identified as the primary chemical mechanism driving irreversible catalyst deactivation.

## 3. Experiment

### 3.1. Catalyst Preparation

The Ru@CoMn_2_O_4_ catalyst was synthesized via the sol–gel method, adopting the procedure detailed in our previous publication [21].

### 3.2. Catalyst Characterization

The crystal structures were characterized using a Bruker D8 Advance X-ray diffractometer (XRD; Cu Kα radiation, 40 kV, 40 mA; Bruker, Ettlingen, Germany). N_2_ adsorption–desorption isotherms were recorded at 77 K using a Quanta chrome AutoSorb iQ-MP analyzer (Quanta chrome, Boca Raton, FL, USA). The Brunauer–Emmett–Teller (BET) method was applied to calculate the specific surface area, and the Barrett-Joyner-Halenda (BJH) model was employed to derive the pore size distribution of the pore. The morphology and crystal structure of the catalysts was analyzed via a JEM-2100F field emission transmission electron microscope (TEM) coupled with energy-dispersive spectroscopy (EDS) for elemental mapping (JEOL Ltd., Tokyo, Japan). Thermogravimetric analysis (TGA) and differential scanning calorimetry (DSC) were employed to investigate the thermal decomposition behavior of the fresh and poisoned catalysts (NETZSCH, Selb, Germany). The analyses were conducted under a high pure N_2_ atmosphere, with a heating rate of 10 °C/min from 25 °C to 1000 °C. The mass spectrometry curves were performed using a Hiden Analytical mass spectrometer (Hiden, Warrington, England). Redox properties were probed by H_2_-TPR and O_2_-TPD experiments using a Quanta chrome ChemStar analyzer (Quanta chrome, Boca Raton, FL, USA) equipped with a thermal conductivity detector. EPR were acquired using a Bruker EMX plus spectrometer (Bruker, Karlsruhe, Germany), operating at X-band microwave frequencies (9.5–12 GHz) with dual-cavity detection. Chemical states of surface elements were investigated by XPS on an AXIS ULTRA DLD spectrometer (Shimadzu, Kyoto, Japan) with a monochromatic Al Kα X-ray source (1486.6 eV).

In situ infrared spectroscopy was utilized to monitor modifications to the catalyst’s surface groups during the catalytic oxidation of propane following SO_2_ introduction. Spectra were recorded on a Tensor 27 infrared spectrometer (Bruker, Karlsruhe, Germany). Prior to measurement, the catalyst was degassed at 300 °C for 1 h under N_2_ to remove adsorbed impurities. Subsequently, a reaction mixture comprising 2000 ppm C_3_H_8_, 30 ppm SO_2_, 10% O_2_, and balanced N_2_ was introduced. The evolution of surface species was then tracked over time. All spectra represent 64 co-added scans acquired at an 8 cm^−1^ resolution, with background subtraction applied.

### 3.3. Catalytic Performance Evaluations

The effect of SO_2_ on the propane removal performance of a Ru@CoMn_2_O_4_ catalyst was investigated in a fixed-bed quartz tubular reactor (inner diameter: 6 mm) operating at atmospheric pressure. The feed gas consisted of 2000 ppm C_3_H_8_, 30 ppm SO_2_, 10% O_2_, and balanced N_2_. Isolating the variable, tests were performed at a constant gas hourly space velocity (GHSV) of 60,000 mL g^−1^ h^−1^ across a temperature range of 200–280 °C, ramping at 5 °C/min with a 0.5 h dwell time at each data point. To elucidate its dynamic role, transient SO_2_ experiments were additionally conducted at 220 °C under identical reaction conditions. Product distributions were analyzed online using a Gasmet DX4000 infrared gas analyzer (Gasmet, Vantaa, Finland).

## 4. Conclusions

This study systematically elucidates the inhibitory effects of SO_2_ on the catalytic oxidation of propane in the Ru@CoMn_2_O_4_ catalyst system using a combination of qualitative and quantitative methods. By comparing the physical and chemical properties of the catalyst before and after intentional SO_2_ poisoning, we identified key degradation mechanisms. Specifically, high valence state Mn (Mn^4+^ and Mn^3+^) active sites were found to undergo conversion to MnSO_4_ upon SO_2_ treatment, accompanied by significant decreases in specific surface area and pore volume. The formation of surface-bound sulfates was shown to block accessible active centers, directly contributing to a measured decline in the catalyst’s redox properties. Although no distinct CoSO_4_ phase was detected after SO_2_ exposure, XPS results revealed partial reduction of Co^3+^ to Co^2+^. The decline in Co’s redox properties was also a significant factor contributing to the reduced propane oxidation activity. Supporting in situ DRIFTS experiments demonstrated that SO_2_ hindered the further oxidation of C=C bonds present in reaction intermediates derived from propane. In summary, propane constitutes a significant fraction of both VOCs and greenhouse gases. Its efficient removal via catalytic oxidation is critically impaired by SO_2_ presence. Revealing the influence of SO_2_ on the catalytic oxidation of low-concentration propane not only clarifies the underlying poisoning mechanisms but also provides guiding principles for the rational design of novel sulfur-tolerant catalysts, thereby fostering advancements in industrial emission control technologies with far-reaching environmental significance.

## Figures and Tables

**Table 1:** The porous structural parameters of fresh and poisoned catalysts.

Catalyst	S_BET_ (m^2^/g)	Average Pore Size (nm)	Pore Volume (cm^3^/g)
Fresh	82.97	17.22	0.36
Poisoned (After SO_2_)	62.69	18.65	0.29

**Table 2:** XPS results of surface Co, Mn, O, S.

Catalyst	Mn^2+^/Mn	Mn^3+^/Mn	Mn^4+^/Mn	Co^2+^/Co	Co^3+^/Co	O_lat_/O_ad_	SO_4_^2−^/SO_3_^2−^
Fresh	0.15	0.58	0.27	0.48	0.52	1.44	--
Poisoned (After SO_2_)	0.36	0.40	0.24	0.58	0.42	1.33	1.72

## Data Availability

Data are contained within the article.
